# Simvastatin impairs the induction of pulmonary fibrosis caused by a western style diet: a preliminary study

**DOI:** 10.1111/jcmm.12637

**Published:** 2015-08-25

**Authors:** Peter Kruzliak, David L Hare, Vaclav Zvonicek, Jan Klimas, Anthony Zulli

**Affiliations:** a International Clinical Research Center, St. Anne's University Hospital and Masaryk UniversityBrno, Czech Republic; b Departments of Cardiology and Medicine, University of Melbourne, Austin HealthMelbourne, VIC, Australia; c Department of Anesthesiology and Intensive Care Medicine, St. Anne's University Hospital and Masaryk UniversityBrno, Czech Republic; d Department of Pharmacology and Toxicology, Faculty of Pharmacy, Comenius UniversityBratislava, Slovakia; e Centre for Chronic Disease Prevention & Management (CCDPM), Western CHRE, College of Health and Biomedicine, Victoria UniversitySt Albans, VIC, Australia

**Keywords:** simvastatin, fibrosis, Hsp70, Hsp90, diet, cholesterol, methionine

## Abstract

The role of an atherogenic diet in causing pulmonary fibrosis has received little attention and simvastatin has been shown to reduce pulmonary fibrosis in animal models. To determine if an atherogenic diet can induce pulmonary fibrosis and whether simvastatin treatment is beneficial by up-regulating heat shock protein 70 and 90. New Zealand white rabbits (*n* = 15) were divided: Group 1 (control); Group 2 (MC) received a normal rabbit diet with 1% methionine plus 0.5% cholesterol (atherogenic diet). Group 3 received the same diet as the MC group plus 5 mg/kg/day simvastatin orally (MCS). After 4 weeks, the lungs were collected and analysed. Picrosirus red staining of lung interstitial collagen content showed that the atherogenic diet increased fibrosis 2.9-fold (*P* < 0.05), bronchiole adventitial collagen was increased 2.3-fold (*P* < 0.05) and bronchiole epithelium was increased 34-fold (*P* < 0.05), and simvastatin treatment severely reduced this effect (*P* < 0.05). Western blot analysis showed that the atherogenic diet significantly reduced lung Hsp70 protein by 22% (*P* < 0.05) and Hsp90 protein by 18% (*P* < 0.05) and simvastatin treatment did not affect this result. However, aortic hyper-responsiveness to vasoconstrictors (angiotensin II and phenylephrine) were markedly reduced by simvastatin treatment. We report that an atherogenic diet stimulates pulmonary fibrosis and reduces lung Hsp70/Hsp90 protein concentration. Simvastatin impairs this by mechanisms unrelated to Hsp70/Hsp90, but possibly a reduction in angiotensin II receptor or alpha adrenergic receptor pathways. These results could have implications in idiopathic pulmonary fibrosis.

## Introduction

Idiopathic pulmonary fibrosis (IPF) is a fibroproliferative disorder, thought to be caused by and unregulated physiological response to microinsults. It is characterized by interstitial alveolar fibrosis and currently, no therapy has proven to reduce this pathology 1. Therefore, the aetiology of the disease and novel therapies are being sought.

The role of a Western diet (high in fat, cholesterol and methionine) in inducing pulmonary fibrosis has received little attention 2 but recent evidence reveals a positive role for diet in the prevention of lung disease. For example, homocysteine, which is formed by the demethylation of the essential amino acid methionine, has a pro-fibrotic role. Elevated plasma homocysteine (tHcy) is associated with increased pulmonary fibrosis in a murine model 3. In a rabbit model, we have shown that a diet high in methionine leads to myocardial fibrosis 4 and clinically, high plasma homocysteine is strongly associated with myocardial fibrosis 5.

Heat shock proteins play a pivotal role in cellular protection. The HSP family are powerful chaperones that help cells cope with increased cellular misfolded and denatured proteins, thus helping cells resist damage and apoptosis. Thus, a decrease in HSPs impairs the ability of a cell to cope with stress, and thus become more prone to apoptosis and inflammation 6–8. Hsp32 (haeme-oxygenase 1) induction has been previously reported to provide protection against pulmonary hypertension in a non-diet murine model 9, and induction of Hsp70 in the rat lung by heat exposure can attenuate lung fibrosis 10, The role of Hsp90 in the regulation of lung fibrosis is not clear, but a recent review suggests that it could be a promoter of cystic fibrosis 11.

It remains unclear if simvastatin treatment can improve pulmonary disease in the clinical setting 12. Cell culture studies on lung fibroblasts show that simvastatin can inhibit the induction of collagen production by transforming growth factor β 13 and studies in mice show that simvastatin can impair bleomycin-induced pulmonary fibrosis 14. In other cell culture studies, simvastatin has been shown to not up-regulate Hsp70 or Hsp90 proteins in osteoblasts 15, but whether or not this effect of statin therapy also extends into dietary-induced pulmonary fibrosis is unclear. Moreover, it is unknown if a protective effect of simvastatin could be because of a reduction in Hsp90 protein.

Furthermore, over stimulation of the renin angiotensin system 16 and alpha adrenergic system 17 also play a major role in pulmonary fibrosis. Finally, we used an atherogenic rabbit model known to induce myocardial fibrosis 4 to study pulmonary fibrosis, and whether simvastatin treatment can ameliorate these alterations *via* changes in lung Hsp70 and Hsp 90 proteins or reactivity to angiotensin II and an alpha adrenergic agonist.

## Materials and methods

### Animals

Male New Zealand White rabbits at 3 months of age (weight range between 2.8 and 3.2 kg) were randomized into 3 groups and fed a specified dietary regimen for 4 weeks, and housed in individual cages and maintained at a constant temperature of approximately 21°C. Food and water were supplied *ad libidum*. The experiments were carried out according to the National Health and Medical Research Council ‘Australian Code of Practice for the Care and Use of Animals for Scientific Purposes’ (6th Edition, 1997).

### Diet

Group (1) control (*n* = 5, Con); Group (2) received a normal rabbit chow diet supplemented 0.5% cholesterol + 1% methionine + 5% peanut oil (*n* = 5, MC) and Group (3) received a normal rabbit chow diet supplemented 0.5% cholesterol + 1% methionine + 5 mg/kg/day simvastatin orally + 5% peanut oil (*n* = 5, MCS).

### Methods

Blood was collected weekly from all animals from the main ear artery and immediately spun at 2236g for 5 min. and the serum collected and snap frozen in liquid nitrogen. The animals were then killed by an overdose intra venous injection of ketamine and xylazine *via* the main ear vein as previously described in our laboratory 18,19. The lungs were then excised and immediately frozen in liquid nitrogen.

### Western blot analysis

Lung samples (15 mg) were collected from 15 rabbits and homogenized in 1 ml lysis buffer (50 mM TrisHCl, pH 8.0, 150 mM NaCl, 1% NP-40, 0.5% Sodium Deoxycholate, 0.1% SDS, 2 mM ethylenediaminetetraacetic acid, 1 mM phenylmethanesulfonyl fluoride, 1/100 Sigma II cocktail, 1 mM NaF and 1 tablet of protease inhibitor cocktail that has been diluted to 1 ml with water). Homogenized tissue was then constantly agitated for 2 hrs at 4°C and centrifuged at 12879g for 5 min. The supernatant was collected, aliquoted and stored at −40°C until used for gel electrophoresis. Acrylamide gels were composed of 8% separating gel overlaid with 5% stacking gel. 30 μg of protein were loaded into each well and electrophoresis was carried out at 100 V for 1 hr and then 180 V for 45 min. Following the electrophoretic separation, the proteins were transferred onto an immune-blot Polyvinylidene Fluoride membrane (15 min. at 15 V). After transfer, the immune-blot PVDF membranes were washed 5 times for 3 min. each in Tris Buffered Saline-Tween and blocked with a blocking solution containing 5% non-fat milk powder in TBS for 45 min. The membranes were then incubated on a rocking platform at slow speed, overnight, at room temperature with Hsp70 (1:1000, cat. no. MAB3516; Millipore, Bayswater, Victoria, Australia), Hsp90 (1:2000, cat. no. 1429-50; Abcam) or α tubulin antibody (1:20,000, cat. no. T6074; Sigma-Aldrich). Then, membranes were washed 5 times for 3 min. each in TBS-Tween and then placed in a blocking solution containing a specific antimouse antibody conjugated to peroxidise (1:7500, cat. no. P0447; Dako). Enhanced chemiluminescence solution was incubated for 1 min. at room temperature on a very slow rocking platform. The bands on the membrane were visualized using the Fuji Film LAS3000, and bands were analysed using FujiFilm MultiGauge Software, Brookvale, NSW, Australia.

### Isometric tension

Thoracic aortae from each rabbit were dissected into 4 × 3 mm rings. The four rings were sequentially mounted between two metal hooks in organ baths attached to force displacement transducers (OB8; Zultek Engineering, Melbourne, Australia).

The baths were filled with Krebs solution and kept at a constant temperature of 37°C and continuously bubbled with 95% O_2_/5% CO_2_. After 1 hr, vessels were gently stretched to a resting tension of 2.5 g. After 15 min., the vessels were gently re-stretched to a resting tension of 2.5 g. After the vessels reached plateau tension, maximum constriction was determined by a high potassium Krebs solution (KPSS, 124 mM K^+^). After plateau (6 min.), vessels were repeatedly rinsed with Krebs solution. After 45 min., the vessel rings were subjected to a phenylephrine concentration curve (10^−8^–10^−5^ M, half log units). After the final concentration of phenylephrine was added and the constriction reached plateau, the vessels were repeatedly washed with Krebs solution and allowed to rest for 1 hr. Then, the vessel rings were subjected to an angiotensin II concentration curve (10^−9^–10^−6^ M, half-log units).

### Immunohistochemistry and collagen stain

Lung biopsies were randomly obtained (4 mm) form all animals and mounted on a single chuck and sections were cut at 5 micron using a cryostat at −20°C. Sections were allowed to dry and then immediately fixed in 4% paraformaldehyde solution in 1× PBS, pH 7.3, for 10 min.

For immunohistochemistry, sections were dehydrated through graded alcohols, delapidated through xylene, and antigenic sites exposed *via* submersion of slides in molten paraffin at 61°C for 30 min. After this, sections were processed through xylene, and rehydrated through alcohol and then water. Sections were placed in 1× PBS for 2 min. and then incubated with 1% goat serum in 1× PBS for 20 min. Then sections were incubated with anti-Hsp70 antibody (1:50) or Hsp90 (1:100) (diluted in 1% donkey serum in 10 mM Tris HCl, pH 7.4) overnight. Immunohistochemistry was then performed as previously described 20,21.

For collagen, pilot experiments were carried out to determine the appropriate concentration and time for staining. Duplicate sections were placed in a 0.001%; 0.01%; 0.1%; 1% picrosirus red solution (dissolved in picric acid) for 30, 45 and 60 min. The final duplicate slides selected were at 0.1% picrosirus red at 45 min. incubation. This allowed for the best contrast between red collagen and yellow background. Sections were washed, dehydrated and mounted with DPX mounting media. Slides were placed under a 20× lens (Olympus microscope, BX53; Macquarie Park, NSW, Australia) and images of interstitium and bronchioles were randomly obtained (2 per biopsy) using a digital camera (Leica DFC490; North Ryde, Australia). The hue, intensity and saturation of the MCID analysis software (MCID Core; InterFocus Imaging, Linton, UK) were adjusted to obtain the red stained collagen, but not the faintly stained red or yellow background. The intensity and proportional area were recorded for collagen, and an average was calculated from all images per animal as published by our laboratory 4. This was then repeated and both results were averaged and the data tabulated.

### Data analysis

All data points were analysed by anova followed by a Dunnets post hoc test. A *P* < 0.05 was accepted in all cases as significant. Vasoconstrictive responses were normalized to KPSS values. All data are expressed as mean ± SEM and normalized to control.

## Results

At the end of the treatment, blood total cholesterol was raised in both MC and MCS groups (9.3 ± 2.9 and 8.8 ± 2.7 mM respectively, *P* < 0.05) *versus* control (1.1 ± 0.2 mM). Low density lipoprotein was raised in MC and MCS (5.5 ± 1.1 and 6.2 ± 2.6 mM respectively, *P* < 0.05) *versus* control (0.32 ± 0.02 mM). Plasma triglycerides were not different between MC, MCS and control (2.2 ± 1.4 mM, 1.8 ± 0.7 mM and 0.6 ± 0.02 mM respectively). Plasma homocysteine was raised in weeks 1–3, but not at the end of the experiment between MC, MCS and control (20 ± 2.1 μM, 33 ± 9.1 μM and 19 ± 1.1 μM respectively).

**Figure 1:**
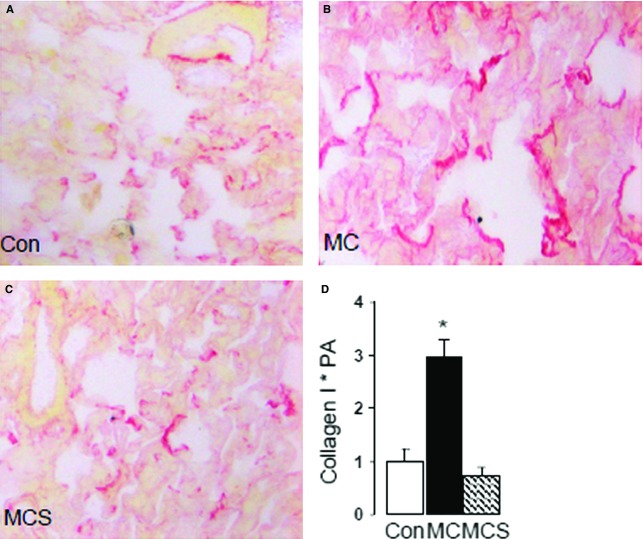
Interstitial fibrosis in the lung indicated by red (sirius red) staining, and background cells by yellow (picric acid). Collagen is observed in the control lung (**A**) and excess collagen deposition in observed in the MC group (**B**), which is restored to control by simvastatin treatment (**C**). Quantification of collagen showed an approximate threefold increase in collagen in MC (**D**, *P < 0.05 *versus* all groups). PA: proportional area.

**Figure 2:**
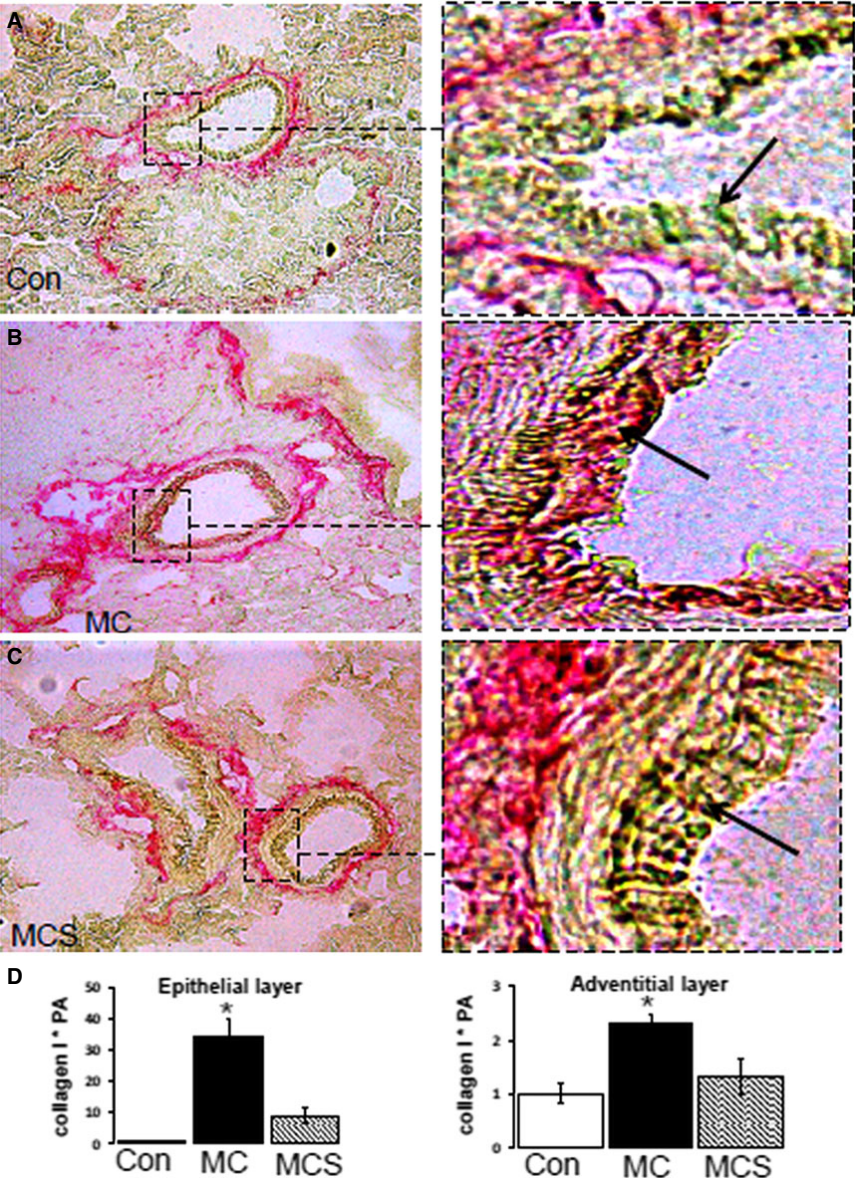
Bronchiole adventitial and epithelial fibrosis in the lung indicated by red (sirius red) staining, and background cells by yellow (picric acid). Collagen is observed in the adventitia but not epithelia of the control lung (**A**) and excess collagen deposition in adventitia and epithelia is observed in the MC group (**B**), which is not observed by simvastatin treatment (**C**). Quantification of collagen (**D**) showed an increase in collagen in MC, and reversed by simvastatin treatment (*P < 0.05).

Analysis of picrosirus red staining of collagen in the pulmonary interstitium (Fig.1A–D) of animals fed the atherogenic diet (MC group) showed a significant increase in pulmonary interstitial collagen (B, D) compared to control (2.9 ± 0.4-fold *versus* 1.00 ± 0.3-fold, *P* < 0.05). Simvastatin treatment inhibited this effect and restored collagen to control levels (0.72 ± 0.25-fold *versus* 1.00 ± 0.3-fold). Bronchiole adventitial collagen (Fig.2A–D) increased 2.3 ± 0.2-fold (*P* < 0.05) and simvastatin reduced this to 1.3 ± 0.32-fold (*P* < 0.05 *versus* MC, *P* = ns *versus* control). Also, bronchiole epithelial cell collagen increased 34 ± 5.5-fold (*P* < 0.05) and simvastatin reduced this to 9.0 ± 2.7-fold (*P* < 0.05 *versus* MC and control).

**Figure 3:**
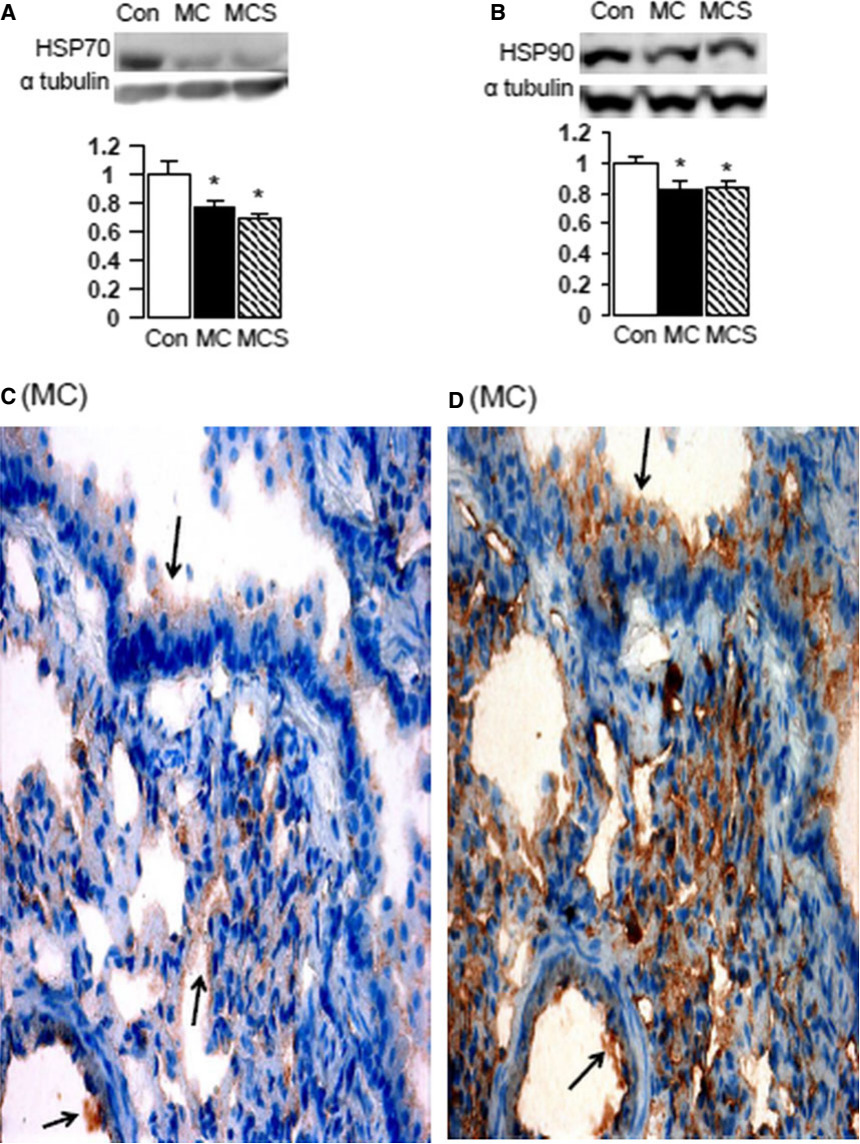
Western blot analysis showed that the atherogenic diet caused a decrease in pulmonary Hsp70 (*P < 0.05 *versus* control, **A**) and Hsp 90 (*P < 0.05 *versus* control, **B**) which was not affected by simvastatin treatment. Immunohistochemistry revealed co-localization (arrows) of Hsp70 (**C**) and Hsp90 (**D**) in the alveolae and endothelial layer of arterioles (×400). Only MC group is shown.

Western blot analysis of both pulmonary Hsp70 and Hsp90 proteins (Fig.3A–D) revealed that the atherogenic diet significantly reduced Hsp70 protein by 23% (A, *P* < 0.05) and Hsp90 protein by 18% (B, *P* < 0.05) compared to control diet. Simvastatin treatment had no effect on pulmonary Hsp70 or Hsp90 (Fig.3A and B). Immunohistochemistry for both Hsp70 and Hsp90 revealed positive co-immunostaining in the endothelium of arterioles and on the surface of alveoli (MC group only shown, Fig.3C and D).

### Vascular reactivity

**Figure 4:**
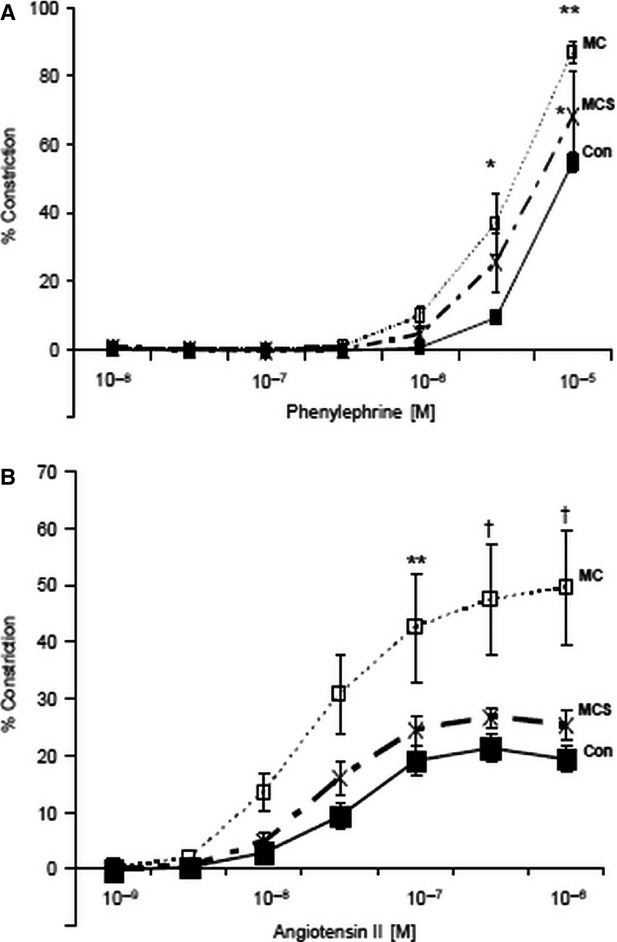
A Simvastatin treatment significantly reduced phenylephrine-induced vasoconstriction compared to MC (*P < 0.05), however, this did not return to control (*P < 0.05). (**B**) Simvastatin treatment significantly reduced angiotensin II-mediated vasoconstriction compared to MC (**P < 0.01, ^†^*P* < 0.001) and this was not significant to control.

After 4 weeks of dietary manipulation (Fig.4A and B), phenylephrine-induced vasoconstriction was also increased in both MC (87.0 ± 3.3%, *P* < 0.001) and MCS groups (68.2 ± 13.4%, *P* < 0.05) *versus* control (54.9 ± 3.0%). Interestingly, the MC group showed increased vasoconstriction to angiotensin II compared to control (49.5 ± 9.9 *versus* 19.4 ± 2.1, *P* < 0.001), which did not occur in the MCS group (25.3 ± 2.6 *versus* 49.5 ± 9.9, *P* < 0.01), the vasoconstriction being virtually normalized by simvastatin treatment when compared to control group (25.3 ± 2.6 *versus* 19.4 ± 2.1, *P* = ns).

## Discussion

The major findings in this study are that (*i*) an atherogenic diet for 4 weeks stimulates the development of pulmonary fibrosis; (*ii*) simvastatin therapy can inhibit this effect regardless of the lipid profile and tHcy; (*iii*) an atherogenic diet reduces the protein expression of pulmonary Hsp70 and 90 and (*iv*) simvastatin does not restore Hsp70 or Hsp90 during an atherogenic diet.

Nutrition is implicated in diverse pathological conditions, namely cardiovascular disease and obesity. The link between diet and pulmonary disease has received little attention 22–24, as exposure to allergens, genetic factors, cigarette smoking are established aeti-ological factors. In our study, we clearly show for the first time that an atherogenic diet designed to mimic excess animal product intake raises plasma LDL and homocysteine which leads to pulmonary interstitial fibrosis. These results have direct implications in the aetiology of IPF.

Inhibition of HMG-CoA reductase (3-hydroxy-3-methyl-glutaryl-Co*A*) by statins is an established treatment for the prevention of cardiovascular disease, and has recently been found to improve exercise capacity in chronic obstructive pulmonary disease patients with pulmonary hypertension 25. The mechanisms might include a beneficial effect on nitric oxide 26,27 and inhibition of endothelin 25. Here, we show that simvastatin can impair the increase in dietary-induced pulmonary fibrosis, an affect clearly not related to either a decrease in plasma cholesterol or homocysteine, indicative of the pleiotropic role of simvastatin therapy. Although statin therapy has been shown to improve exercise capacity in humans, the mechanisms remain elusive – whether simvastatin stimulates the remodelling of pulmonary structure to resemble ‘normal’ collagen content remains to be elucidated. Indeed, it is established that statin treatment can reduce Akt signalling 28 and suppress inflammation 29, proposing that suppression of inflammatory signalling could reduce collagen accumulation. Furthermore, recent studies associate statin use with the induction of pulmonary fibrosis 30. Here we show that simvastatin can reduce the induction of pulmonary fibrosis caused by an atherogenic diet, and these effects of statin therapy on pulmonary fibrosis are supported in other models 31–33. Thus, further studies aimed at understanding the mechanisms involved with simvastatin use in pulmonary fibrosis are warranted.

The atherogenic diet caused a significant reduction in Hsp70 and Hsp90, which was associated with the increase in pulmonary collagen content. Whether a direct causal relationship exists between a reduction in Hsp70/Hsp90 and pulmonary fibrosis is unclear. Although an increase in Hsp70 has been shown to protect rats from pulmonary fibrosis 10, the opposite might not hold true. Indeed, simvastatin therapy inhibited dietary-induced pulmonary fibrosis without affecting both Hsp70/Hsp90, clearly indicating that the reduction in these two proteins might not be causing the fibrosis observed. Moreover, Hsp70 has been suggested to be a sensitive marker for oxidative stress in arteries 34 and we have observed an increase in Hsp70 in the coronary artery of rabbits fed a similar diet 20. We propose that the atherogenic diet is impeding the signalling pathways which stimulate the up-regulation of Hsp70 in the lung. Further research understanding the signalling pathways linked to Hsp70 up-regulation will shed light on this phenomenon.

Hsp90 has been recently identified as a possible promoter of collagen deposition 11, suggesting that a decrease in this protein should prevent the accumulation of collagen. We show that the atherogenic diet does reduce Hsp90, possibly in an attempt to reduce collagen deposition even though excess collagen deposition occurred in this model. Further studies aimed at reducing Hsp90 protein could help elucidate the role of Hsp90 in dietary-induced pulmonary fibrosis. As collagen accumulation was impaired by simvastatin treatment regardless of Hsp90 protein levels, studies aimed at determining Hsp90 protein activity in this model could further aid in understanding the role for Hsp90 in collagen deposition.

Simvastatin has pleiotropic effects on endothelial nitric oxide synthase (eNOS). We have currently shown that in these same animals, simvastatin impaired the ability of the atherogenic diet to cause endothelial dysfunction in blood vessels, and this was not associated with an increase in aortic endothelial eNOS, changes in the phosphorylation of eNOS at the threonine 495 site or serine 1177, but simvastatin treatment significantly reduced endothelial caveolin-1 by 35% 35. Considering an increase in caveolin-1 has been linked to pulmonary fibrosis 36, studies in this model addressing the role of caveolin-1 in the cause and reduction of pulmonary fibrosis in this model is warranted.

Interestingly, we found a reduction in angiotensin II-mediated vasoconstriction in the aorta of the animals fed the atherogenic diet treated with simvastatin. The role of angiotensin II in mediating pulmonary fibrosis is well-established 37,38, and the effect of simvastatin in reducing angiotensin II-mediated signalling is well reported 39–41. Also, we found a reduction in alpha adrenergic-mediated vasoconstriction (using phenylpherine) in the aorta of the animals fed the atherogenic diet treated with simvastatin, but this did not return to normal. Also, the role of adrenergic mechanisms in the pathogenesis of fibrosis is being recognized 17 and the effect of simvastatin on reducing adrenergic mechanisms is reported 42. Thus, in this model, it is possible that simvastatin treatment is reducing both angiotensin II and adrenergic mechanism-mediated fibrosis. Further studies aimed at establishing this link in pulmonary fibrosis will help establish whether or not angiotensin II receptor blockers, adrenergic blockers and/or statin therapy can be used to treat this condition.

In conclusion, we report that an atherogenic diet for 4 weeks can increase pulmonary fibrosis in the rabbit, which is associated with reduction in Hsp70 and Hsp90. We also show that simvastatin impairs pulmonary fibrosis without affecting plasma lipids, tHcy levels, or Hsp70 and Hsp90, but reduces angiotensin II and phenylephrine-mediated aortic vasoconstriction. These results have direct implications in the aetiology and treatment of IPF.
